# On the Path to Global Open Access: A Few More Miles to Go

**DOI:** 10.1371/journal.pmed.1001014

**Published:** 2011-03-29

**Authors:** The PLoS Medicine Editors

## Abstract

The *PLoS Medicine* editors reflect on the recent debate about access to medical journals via HINARI, and what now needs to be done to increase open access in the developing world.

It has been a couple of months now since the withdrawal of access via HINARI to medical journals in Bangladesh by several publishers caused an upset in the medical publishing world [1]. HINARI (Health Internetwork Access to Research Initiative) is a WHO-supported program [2] that partners with subscription-based publishers to allow researchers in the world's poorest countries to access some of their journals under certain conditions (for example, researchers have to access the journal in defined institutions). After much lobbying from researchers, editors, and others following the withdrawal, HINARI access has been—for the time being at least—reinstated, though with a substantial lack of clarity over the longer term plans of a number of the publishers [3]. Although traumatic for the researchers who lost access, the incident has triggered a useful debate on the value of open access (OA; immediate, permanent free access and permanently guaranteed unrestricted reuse, as enshrined in a Creative Commons license [4] and as practiced by publishers such as PLoS) versus free access with no legal rights attached. It is hard to think of a better example to demonstrate the precariousness of this latter type of free access, which can mean that access may be withdrawn for no reason.

Now that the heat of the HINARI debate has died down, it is an opportune time to consider how this dispute, and others like it, can be used constructively to move toward a position where universal OA to the medical literature becomes the norm.

On the positive side, the debate has brought many new voices into the discussion around access, particularly those on the online discussion forum HIFA2015 [5], where the diversity and strength of opinions expressed was most likely the key instrument in ensuring that the publishers' withdrawal from HINARI was not only brought to light, but also largely reversed.

The debacle also allowed constructive discussions around the substantial limitations of HINARI and its inability to provide a long-term sustainable solution to access in the developing world. It also allowed airing of many OA issues, including the difference between free and open access [4]; the logistical difficulties experienced by some researchers in accessing online journals, such as those in locations with low bandwidth; the suspicion of some researchers of online-only journals; and concerns over publication fees.

Thus the argument about how to implement such access, particularly in the developing world, is far from over. The issues above are very familiar to OA advocates. When *PLoS Medicine* was getting started seven years ago, we encountered many of the same questions from the (admittedly mostly developed-world) authors and readers we canvassed then. The phenomenal growth of OA since then has reassured many of those who initially questioned the model and its sustainability: submissions and publications are increasing each year at PLoS and in other open-access journals, reflecting the increased confidence of authors in this model. OA papers are also highly accessed, though our data suggest that most of this access, and most of the authors, still come from the developed world.

The HINARI incident thus highlights the fact that HINARI is, sadly, still needed both because of traditional publishers who have not yet implemented OA, even in the developed world, and because substantial gaps remain in our knowledge about how OA will work for the developing world. Hence, there is some way to go before this model of publishing can become the norm worldwide. Despite the best intentions of open-access publishers, we have failed to reach out adequately to debate with researchers and readers in the less-developed world about the potential benefits of open access. Instead, as is often the case when the developed world prescribes for the less-developed world, we have assumed that what works well in Paris, London, or San Francisco will work just as well in Addis Ababa, Beirut, or Lima.

Some examples of these active concerns about OA: first, are OA journals being delivered in the best format for readers in the developing world? If print really is better in some places, are we doing our best to ensure that the online journals are optimized for rapid downloading and printing of articles? If access to online journals will be primarily via mobile devices rather than computers, are we delivering the content in appropriate formats? Second, do we understand the reputation metrics outside of Europe or the US that will ensure that the new OA journals are trusted and meet the requirements authors face for academic promotions or other professional needs [6]? Even more importantly, are there OA journals available that cater to the needs of readers and authors across the developing world? Should publishers be helping groups to start their own journals, rather than assuming that the existing OA journals will be accepted?

Medical journals have many roles, but, above all, dissemination of medical information is key. This crucial role was stated clearly back in1997 by Neil Pakenham-Walsh (the founder of HIFA2015) and colleagues, and it is no less relevant now [7]: “Providing access to reliable health information for health workers in developing countries is potentially the single most cost effective and achievable strategy for sustainable improvement in health care.”

Much therefore remains to be done in improving access to health information in the developing world. By providing a logistical framework for open access (by the adoption of appropriate licenses), and by showing what can be done in the developed world with OA journals, OA publishers have done much to make it possible more widely. The next crucial step is to engage with readers, researchers, and authors in the developing world to understand better their information needs so that we don't fall into the trap of pushing information in only one direction. Open access is about facilitating the movement of knowledge—in all directions.

## Abbreviations

HINARI: Health Internetwork Access to Research Initiative
OA: open access
